# Address, Delivered before the Mississippi Valley Association of Dental Surgeons

**Published:** 1844-12

**Authors:** John Allen


### ARTICLE III.

*Address*, delivered before the Mississippi Valley Association of Dental Surgeons, at the Ohio Medical College in Cincinnati, August 14th, 1844. By Dr. John Allen.

To ameliorate the condition of man, has ever been considered, by all civilized nations, as an object of great importance, and we find that the most brilliant minds, in every sphere of life, have made this an object of their highest ambition.

The honest statesman directs the energies of his mind to the enactment of such laws, the cherishing of such sentiments, and the fostering of such institutions, as will secure this end. The divine pours forth, in continued strains of eloquence, the doctrines and principles which, in his opinion, secures this boon to man while here on earth, and leads to eternal bliss in the world to come.

It is to ameliorate the condition of man that the patriot buckles on his armor, and unsheathes his sword, to sever the bonds of oppression.

For this purpose the physician qualifies himself for the discharge of those high and responsible duties which devolve upon him, by which he is enabled to alleviate the thousand ills of life with which man's cup is filled, and to pour in the oil of gladness, which, but for his aid, must have continued to overflow with misery. It is for this, too, that the judicious and well qualified dentist, by the combined agency of science and art, contributes largely to the common stock by mitigating the bitter pangs of human suffering.

Dental surgery has long been considered, by the most enlightened nations, an important branch of the healing art: yet there are many who suppose it to be of recent origin: that formerly, diseases of the mouth did not, as at present, exist; perhaps, to the same extent, they did not; but we find, in some of the most ancient historical works, that the care of the teeth was assigned to a particular class; but, in reference to the extent of their anatomical, physiological, or pathological information upon the subject, we are not informed; they seem to have had some crude ideas of the structure and functions of the teeth.

To trace the progress of any branch of science in which we feel interested, is not only gratifying, but it enables us to judge more accurately of the relative merits of the various theories with which we may become acquainted, thereby enabling us to arrive at a more condensed and practical knowledge of the subject to which our attention may be directed. In order, therefore, that we may be able to give a full and comprehensive view of dental surgery, we will, in the first place, take a hasty review of the past by commencing far back to some of the first buddings of this important branch of the healing art, and trace it along through its various meanderings down to the present time.

One of the first and most distinguished writers upon this subject was Hippocrates, who was born in the island of Cos, about 460 years before Christ; his medical reputation, at the time he wrote, had never been surpassed; and, even now, he is acknowledged by many to have been the father of medical science.

He devoted much attention to the subject of the teeth, and urged the importance of especial care to their preservation; he tells us that, then the teeth were subject to decay, and, consequently, to loss; and that he supplied them with artificial ones, made of bone and ivory. He sometimes used natural teeth, secured by means of ligatures made of flax, silk, and gold or silver wire. Then we have the writings of Aristotle, who tells us that man has more teeth than woman; and of Homer, who thought the teeth were little barriers opposed by nature to the unruliness of the tongue, and the abuse of speech thereby designating them as forming a wall or fence, as a kind of fortification for the tongue, and to carry out the idea, the more teeth that surrounded it, the more strongly it was fortified, and, consequently, less liable to frequent and unwarrantable outbreaks.

This we would not call very sound philosophy, yet we must recollect that this ray of light, however much it may become dimmed by subsequent experience, emanated from one of the most brilliant stars of antiquity; and we find that, for the next three or four hundred years, very little light was thrown upon the subject; which inference we draw from the fact, that about that time Aretenese, who was then acknowledged to be the most learned upon the subject, tells us, that with all his wisdom and philosophy, he was utterly unable to account for the tooth-ache.

About 150 years after Christ, Galen, whose life was spent in the zealous pursuit of knowledge, and, especially every thing connected with the science of medicine, is said to have written about seven hundred and fifty different essays on such subjects, and it is said, that the splendor of Galen's talents so dazzled his successors, that for sixteen hundred years, his opinions bore almost undivided sway. He wrote a much better work upon the teeth than any of his predecessors. He tells us that the teeth are formed during gestation.

He gives a very correct anatomical description of them; he says, they are possessed of nerves which admit of acute sensation, &c., &c.

Soon after, another distinguished writer appeared, whose name was Ætius; who thought the teeth were open at the roots, where they admit several nerves coming from the trifacial; and that they are the only bones that can become painful; he says, they continue to grow till old age, by a deposition in their interior, of a nervous fluid, but at that period, the process of nutrition ceases, and they become loose and fall out. Now, I think, that however correct his theory might have been considered at that time, were he now living, he would find that many teeth become loose and drop out, long before old age brings them out; but as we proceed on down the course of time, we shall pass over the days of Rhazes, Albucasis, Vesalius, Eustachius, and others, whose writings shed but little light upon our path.

This brings us down to the year 1579, in which Paré, a celebrated French surgeon, wrote a very correct essay upon the teeth, although differing in many points from his predecessors and successors, he enjoyed a high medical reputation, and was appointed surgeon in ordinary to Henry II., which office he held under three succeeding kings; he was the author of several popular works which were universally approved, and translated into most of the languages of Europe, containing volumes of medical science; he was a bold and successful operator, and displayed, on many occasions, the talent and skill of an enlightened surgeon; he considered that the adhesion of the teeth to the jaw was strengthened by a ligament which extends from the root of the tooth to the socket; he thought with Ætius, that the teeth continued to grow while they remain sound in the mouth, for, otherwise, by their continual use in masticating, they would be worn and wasted away by one another. His cure for the tooth-ache was to thrust a hot wire into the roots, or by introducing a pledget of cotton dipt in oil of vitriol.

Upon the subject of artificial teeth, he says they should be made of bone or ivory, and that they should be joined together, and then fastened to the adjoining teeth; he also recommended the insertion of human teeth, to be secured by a thread of silk or flax, or gold or silver wire; thus you see that Paré, in A. D. 1579, adopted the same method of inserting artificial teeth that Hippocrates employed four hundred years before Christ. This shows the slow march of improvement in our profession during a period of more than 1800 years.

From 1579 to 1778, at which time Hunter wrote, we get but little additional information, and yet we have the writings of more than one hundred different authors upon this subject during that period. Hunter enters into the subject in a very minute and scientific manner; he shows, very clearly, the organization of the teeth, and points out some of the best means of regulating and of preserving them from decay. Yet high as this author stands in his profession, still there are some discrepancies of opinion between him and other subsequent writers, and practitioners of the dental art; some of his operations that were most objectionable, were in the use of the actual and other cauteries to exposed nerves in decayed teeth; taking out decayed teeth and boiling them, and then replacing them in the mouth; burning the ear with a hot iron, &c. He also sanctioned and advocated the practice of transplanting the teeth, as taught by Paré 200 years before.

The next most eminent writer after Hunter was Bichat, who wrote one of the best works of the age upon that subject. Blandin, a colleague of Bichat, was instrumental in throwing a flood of light upon the subject of the teeth.

This brings us down to A. D. 1801, when Dr. Blake wrote a treatise, which will ever be regarded as a standard work; he was one of those few authors who learned his lessons from nature, and his deductions were all drawn from observation and experience, and were practically useful.

His views respecting the crusta petrosa were original, and have since been approved very generally.

Next we have the work of Fox of London, which appeared in A. D. 1803; he was not only a sound theorist, and able lecturer, but also one of the best practitioners the world had ever produced.

Fox discovered the vascularity of the teeth, and says the investing membrane derives its vessels from the gums, and the pulp from the jaw. Upon this point he differs from some of his most able predecessors, viz. Hunter and Blake. Since Fox, we have the works of Koecker, Jobson, Wait, Rouseaus, Serres, Bell, Audibran, Legros, Lemair, Marmont, Maury, Perland, Ricci, Stunberg, Troubat, Trenor, Fitch, Clark, Murry, Fay, and many others, whose writings have reflected much light upon our profession.

Having taken a hasty and imperfect view of the past, we find that dentists have long been considered an important class of operators, and many of the authors who have written upon the subject have been among the most distinguished men of the age in which they lived, and it is a matter of congratulation that we live in an age and country where we have the advantages which are to be derived from the experience of the most eminent operators for the last twenty-two hundred years. Thus, century upon century has been rolling on, and pouring in tributaries to our aid, from which we are enabled to draw correct conclusions from the experience of those who have gone before us, to lop off the excrescences, if any there be, and to treasure up and retain that which is good, this is our duty; we should qualify ourselves for the task.

Although much has been done, yet much remains to be done, for there are still false theories to be cleared away, with which our profession is loaded, greater eminence among us must be attained, empiricism must be expelled, public confidence must be won, and the line between competent and incompetent operators must be drawn, in order to accomplish the object of our design; the necessity for this will readily be seen when we look abroad upon society, and see the great amount of deformity exhibited, from a want of well arranged teeth, and the thousands of teeth that are crumbling away by decay, from want of proper attention, and when we see that once brilliant orator shorn of his former eloquence, his voice flat and insipid, and his speech rendered powerless from the loss of those hitherto beautiful organs, which gave fullness to the voice, and clearness to the enunciation; when we behold that once beautiful form, upon which nature had lavished her proudest gifts, now exhibiting a mouth rendered loathsome by decay and loss of teeth; when we see that lovely boy, who once gave fair promise of future eminence and renown, disappointed in his high expectations by the malformation of his dental arch, which is now filled only with an irregular, zig-zag and hideous set of teeth, which cause an indistinct, mongrel speech, with harsh and discordant sounds, instead of the well developed maxillaries, adorned with those important embellishments which give beauty of form, symmetry in appearance, expression to the countenance, distinctness to the articulation, richness, force and melody to the voice. When we look at these, in connection with the long train of maladies which are induced by an unhealthy condition of the mouth, we are ready to exclaim, is there no help for all these evils? Are there no competent dentists in the land? Yes, there are remedies which, if properly applied, under favorable circumstances, will as certainly prevent the evils referred to, as that to-morrow's sun will shine, if there be a cloudless sky; and there are dentists who are well qualified for the task, and have the means in their power of diverting these unwelcome messengers of misery from their wonted course. Then why is it not done? One of the most prominent reasons is, that the great masses of the community are not aware that it can be done. There is a great want of confidence in dental operations, and in those who perform them, consequently, a mere modicum of the dental business that the people require is done.

This arises from the fact, that general information upon this subject has not been sufficiently diffused, its genial rays have not been reflected upon society, it is, therefore, perfectly natural for incredulity and prejudice to prevail in the absence of correct information. This want of confidence has been perpetuated by many, very many incompetent operators, who have been constantly traversing the country, who seem to be actuated more by sinister motives than the promotion of the public good, or the elevation of the dental profession. The effect of this has been to confirm the prejudices and preconceived opinions that formerly existed, and form a barrier to the advancement of the dental science. It is for the purpose of breaking down this barrier, removing this prejudice from the public mind, and elevating the standard of our profession, that we have assembled in council upon this occasion, to put forth a united effort in the adoption of such measures as will be most likely to accomplish these objects, for it will be recollected that harmony and concert of action, are always necessary for the accomplishment of important and honorable designs. It is manifest that men can do more good, and accomplish more important objects, when formed into well regulated societies than it would be possible for them to achieve in an individual capacity, hence the establishment of churches, colleges and other halls of science and learning, national and state legislation, and all bodies, politic and corporate. But, hitherto, in this great and fertile valley of the Mississippi, we have not put forth a united effort to elevate the standard of our profession, and place it upon an equal footing with the other professions, so that a generous community might look upon it with that respect and confidence which it deserves. No, we have come into the field, single-handed, and with a kind of secret, selfish tenacity, have put our light (if we had any) under a bushel; we have not permitted it to shine forth upon the people that they might see the benefits, and appreciate the advantages which are to be derived from well performed dental operations. In order, therefore, to promote the cause of dental science, we must gather together the materials of which it is composed, and by union and concert of action, we must raise the standard, and place our mark high upon it, we must, by the sun of science, dispel the dark clouds of ignorance, superstition and prejudice that prevail in the community upon this subject, we must let our light so shine as to reflect its genial influence upon society, and honor upon our profession. Then success will crown our efforts, and mutual advantage redound to all.

### ARTICLE IV.

*Proceedings of a Convention of Professional Dentists.* Held August 13th and 14th, 1844, in Cincinnati.

